# 

**Published:** 1888-05

**Authors:** 


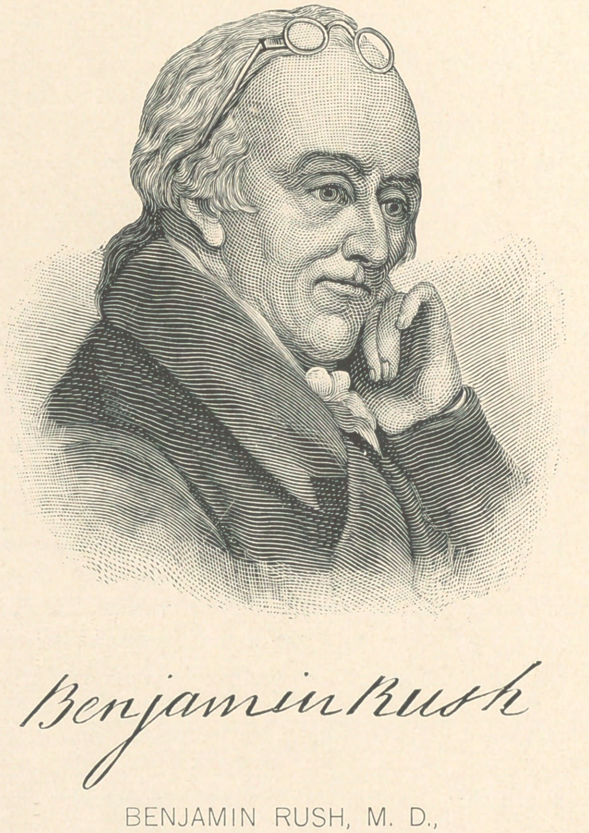

BENJAMIN RUSH, M. D.,

A GREAT PHYSICIAN, A NOBLE PATRIOT, A SIGNER OF THE DECLARATION OF INDEPENDENCE.

### DR. BENJAMIN RUSH.

A wise physician, patriot, Christian gentleman, and a cultured writer—such was Dr. Benjamin Rush.

The elder of two brothers, he was born in the township of Byberry, near Philadeldelphia, and having lost his father in early life, he owed to a devoted mother a generous education.

At the age of fourteen he entered Princeton College, and September, 1760, was graduated with the degree of Bachelor of Arts.

After his graduation he took up the study of medicine under the preceptorship of Dr. Redman, of Philadelphia.

After a pupilage of six years, he went, in 1766, to Edinburgh, where he attended lectures at the university, obtaining, at the end of two years, his degree as Doctor of Medicine. The following winter was spent in London, and the succeeding summer in Paris, and in the autumn of 1769 he returned to Philadelphia, and began the practice of his profession.

He pursued his practice with success, and in the year 1793 a terrible epidemic of yellow fever visited the city. The epidemic broke out during the first week of August, and raged with singular virulence until the end of October, and during that period the city was nearly depopulated. The inhabitants fled panic stricken. In the city alone, the disease counted about 4,000 victims.

Dr. Rush never wavered in his duty. At one time, when 6,000 people were down with the disease, only he and two other physicians stood to their posts, the rest having, it is stated, either died or removed to places of safety.

For weeks, day and night, his house was filled and surrounded by multitudes imploring his assistance. In fact, he nearly paid for his labors with his own life, naturally succumbing at last to a strain that no human system could endure.

His treatment was "heroic." Indeed, Dr. Rush was no believer in what has been aptly described as "long range practice." But though his practice was successful, it aroused a controversy that degenerated into malicious and scurrilous personal abuse. But the world recognized his greatness. Honors and presents flowed in upon him from all countries, and among those gifts was a valuable one from the Emperor of Russia.

The history of Dr. Rush as a teacher of medicine is familiar; his appointments, as chronicled in the journal of the University of Pennsylvania, were as follows: 1769, Professor of Chemistry in the College of Philadelphia; 1789, Theory and Practice of Medicine in the same institution, succeeding Dr. Morgan; 1791, Institutes of Medicine and Clinical Science in the University of Pennsylvania, into which the College of Philadelphia had been merged; 1796, Practice of Physics; this in addition to the last-mentioned chairs.

He was, besides, during many years, one of the physicians of the Pennsylvania Hospital.

He was a thoughtful and polished writer on general subjects, and some of his ideas on non-medical matters, especially that of general education, have since borne fruit.

In July, 1776, he was chosen a representative to the General Congress, and signed his name to the Declaration of Indepence.

In 1777 he was appointed for the Middle Department, Physician-General of Military Hospitals in the Continental Army, and in 1780 he became a member of the delegation from Pennsylvania to the convention that adopted the Federal Constitution.

On the close of the war, he was appointed to the directorship of the Mint, which position he filled during fourteen years.

In private life, he was an active promoter of various social enterprises, being Vice-President of the Philadelphia Bible Society ; Vice-President of the American Philosophical Society, etc. He was also for a time President of the Philadelphia Medical Society. In 1786 he instituted the Philadelphia Dispensary, and was active in founding Dickinson College, at Carlisle, Pennsylvania, as well as an earnest advocate of a free-school system.

These facts serve to illustrate the breadth and liberality of his views, which were far in advance of his times.

He died of typhus fever, in the height of his intellectual vigor and fame, on the 19th of April, 1813, in his 68th year, honored, respected, and beloved. His loss was more deeply lamented throughout the country than that of any other man of his day excepting Washington and Franklin. He left many contributions to the literature of his profession, and though his medical writings have been superseded, his influence upon the methods of the profession remains unimpaired.

In his native city, the magnificent Rush Library, on South Broad street, stands today a monument to his fame and his generosity; and his name is still respected after a lapse of nearly four score years, as that of a hero, gentlemen, scholar, and Christian of the noblest type.

### PAMPHLETS RECEIVED.

Transactions of the American Dermatological Association. Eleventh Annual Meeting, 1887.

Syphilis of the Endometrium. By T. A. Ashley, M. D.

The Galvano-Cautery Sound and its Application, Especially in Hypertrophy of the Prostate. By Robert Newman, M. D.

Synopsis of the Second Hundred Cases of Urethral Stricture, Treated by Electrolysis, with cases. By Robert Newman, M. D.

The Use of the Curette for the Relief of Hæmorrhage Due to Uterine Fibrods. By Henry C. Coe, M. D.

The Significance of Localization of Pain in Pelvic Disease. By Henry C. Coe, M. D.

Dangers in Gasoline. By John H. Kellogg, M. D.

First Quarterly Report of the Michigan State Laboratory of Hygiene. By Victor C. Vaughan, M.D., Ph. D.

The New York Medical Journal Visiting-List and Complete Pocket Account Book.

The Cure of Hernia. By Henry O. Marcy, A. M., M. D., LL. D.

Cystitis in the Female. By Henry O. Marcy, A. M., M. D., LL. D.

On the Use of the Vaginal Tampon in the Treatment of Certain Effects Following Pelvic Inflammations. By Thomas Addis Emmet, M. D

Results of Observation in the Treatment of 2,072 Cases of Lateral Curvature of the Spine. By James Knight, M. D.

A Very Valuable Lesson for Those who Use Anæsthetics. By Julian J. Chisholm, M. D.

Are Dipsomania, Kleptomania, Pyromania, etc., Varied Forms of Mental Disease? By Orpheus Evarts, M. D.

The Lamb Prize Essays:

No. 1. Healthy Homes and Foods for the Working Classes. By Victor C. Vaughan, M. D., Ph. D.

No. 2. The Sanitary Conditions and Necessities of School-Houses and School-Life. By D. F. Lincoln, M. D.

No. 3. Disinfection and Individual Prophylaxis Against Infectious Diseases. By George M. Sternberg, M. D.

No. 4. The Preventable Causes of Disease, Injury, and Death in American Manufactories and Workshops, etc. By George H. Ireland.

### ANNOUNCEMENTS.

### ILLINOIS STATE MEDICAL SOCIETY.

The thirty-eighth annual meeting will be held in Rock Island, in the Opera House, beginning on Tuesday, May 15, 1888, at 10 o'clock, a. m.

OFFICERS OF THE SOCIETY.—President: William Oren Ensign, Rutland. First Vice-President: Charles Warrington Earle, Chicago. Second Vice-President: Phillip H. Oyler, Mt. Pulaski. Permanent Secretary: David W. Graham, Chicago. Assistant Secretary: George L. Eyster, Rock Island. Treasurer: Walter Hay, Chicago.

JUDICIAL COUNCIL.—Ephraim Ingals, Chicago; Francis B. Haller, Vandalia; William Hill, Bloomington; whose term expires 1888. Charles C. Hunt, Dixon; A. T. Barnes, Bloomington; L. G. Thompson,

Lacon; whose term expires 1889. Alphonzo Wetmore, Waterloo; Samuel G. Plummer, Rock Island; Benjamin F. Crummer, Warren; whose term expires 1890.

STANDING COMMITTEES.—Practice of Medicine: Frank Billings, Chicago; G. W. Nesbitt, Sycamore; Adelbert H. Tagert, Chicago. Surgery: W. S. Caldwell, Freeport; Benjamin F. Crummer, Warren; Samuel K. Crawford, Chicago. Obstetrics: Geo. Wheeler Jones, Danville; Charles C. Hunt, Dixon; Catherine Slater, Aurora. Gynæcology: A. Reeves Jackson, Chicago; T. M. Cullimore, Jacksonville; Mary E. Bates, Chicago. Drugs and Medicines: D. S. Booth, Sparta; A. J. C. Saunier, Libertyville; John A. Robison, Chicago. Ophthalmology and Otology: Edward L. Holmes, Seth S. Bishop, Chicago; C. A. Palmer, Princeton. Necrology: Ephraim Ingals, Chicago; John H. Rauch, Springfield; Otho B. Will, Peoria. Publication: David W. Graham, Walter Hay, Chicago; George L. Eyster, Rock Island. Committee of Arrangements: G. Truesdale, Thomas Galt, C. Bernhardi, G. G. Craig, S. C. Plummer, C. C. Carter, Geo. E. Barth, P. Gregg, G. L. Eyster (ex-officio), Rock Island.

SPECIAL COMMITTEES.—Diseases of Children: A. E. Goodwin, Rockford; Mary H. Thompson, Chicago; Washington West, Belleville. Antiseptic Obstetrics: Chas. W. Earle, Chicago. Intubation of Larnyx: Frank E. Waxham, Chicago. The Present Status of Bacteriology in its Relation to Disease: Romaine J. Curtis, Joliet; Frank S. Johnson, Chicago. Pelvic Surgery: Wm. S. Chenoweth, Decatur; Sarah H. Stevenson, Chicago. Venereal Disease: Harold N. Moyer, Isaac N. Danforth, J. J. M. Angear, Chicago. Hypnotics: Daniel R. Brower, Chicago. Compound Fractures: D. A. K. Steele, Chicago. Hernia: David W. Graham, Chicago. Some Topic on Gynæcology: Marie J. Mergler, Chicago. Physiology: Alphonzo Wetmore, Waterloo. Dermatology: Henry J. Reynolds, Chicago. Neurology: Horace Wardner, Anna; J. L. Gray, Chicago. The Exigencies and Excitements During the First Labor: James S. Whitmire, Metamora. Legislation for the Insane: Walter Hay, Chicago; Edgar P. Cook, Mendota; Francis B. Haller, Vandalia. Medical and Sanitary Legislation: B. M. Griffith, Springfield; W. A. Haskell, Alton; John L. White, Bloomington; Albert B. Strong, Chicago. Influence of Appreciable Meteorological and Topographical Conditions on the Prevalence of Acute Diseases: Nathan S. Davis, John H. Hollister, James F. Todd, Chicago; Edgar P. Cook, Mendota; George W. Jones, Danville. Biographical: John H. Hollister, Ephraim Ingals, Chicago; Charles C. Hunt, Dixon; Francis B. Haller, Vandalia; A. M. Powell, Collinsville; Edgar P. Cook, Mendota; John S. Williams, Otterville; George W. Jones, Danville; Thomas F. Worrell, Bloomington; Robert Boal, Peoria; Hugh R. Guthrie, Sparta. Improvement of State and Local Medical Organization, and on Revision of the Constitution: Edgar P. Cook, Mendota; Thomas M. McIlvaine, Peoria; A. E. Goodwin, Rockford. Appointment of Delegates to the American Medical Association for 1888: Charles W. Earle, David W. Graham, Chicago; Geo. W. Jones, Danville.

The committee of arrangements has secured reduction of railroad fare for members and delegates on the conditions specified in circular.

The constitution of the society requires all members and delegates to register and present their credentials before participating in any business of the meeting.

Registration will begin at 9 o'clock a. m., on Tuesday, at the hall. It is desired that as many as can do so will register before the society is called to order.

D. W. GRAHAM,
*Permanent Sec'y*, 133 *Clark St.*, *Chicago.*

NOTICE.—Members who wish to go to the meeting of the American Medical

Association, in Cincinnati, May 8, 1888, as delegates from this society, should send their names to the secretary at once.

C. W. Earle,
D. W. Graham,
G. W. Jones,
*Committee.*

---

Preliminary Programme of the American Association of Genito-Urinary Surgeons—At the meeting to be held in Washington, September 18, 19, and 20, 1888.

1. Clinical Observations on Diseases of Testicle. By Dr. L. B. Bangs, of New York City, New York.

2. Clinical Observations on Chronic Gonorrhœa, and

3. Two Cases of Cancer of the Seminal Vesicles, with Pathological Specimens. By Dr. J. P. Bryson, of St. Louis, Mo.

4. Operative Treatment of Hypertrophy of the Prostate, and

5. Case of Bowel ending in the Urethra of a child four weeks old; Relief by Operation. By Dr. A. T. Cabot, of Boston, Mass.

6. On the Effects of Rapid Changes of Altitude in an advanced Case of Interstitial Nephritis. By Dr. George Chismore, of San Francisco, Cal.

7. Connection between Masturbation and Stricture. By Dr. S. W. Gross, of Philadelphia, Pa.

8. Operations on the Kidney. By Dr. W. H. Hingston, of Montreal, Canada.

9. Syphiloma of the Vulva. By Dr. J. N. Hyde, of Chicago, Ill.

10. The Curability of Urethral Stricture by Electricity; an Investigation, and

11. The Comparative Value of Supra Pubic and Perineal Drainage in Curable and Incurable Bladder Disease. By Dr. E. L. Keyes, of New York City, N. Y.

12. The Filaria Sanguinis Hominis, in the United States, Especially in its Relationship to Chylocele of the Tunica Vaginalis Testis. By Dr. W. M. Mastin, of Mobile, Ala.

13. A case of Perineal Section for Traumatic Retention; Unusual Condition of the Bladder. By Dr. J. E. Michael, of Baltimore, Md.

14. The Prophylaxis of Syphilis. By Dr. P. A. Morrow, of New York City, N. Y.

15. Unusual Case of Urethral Calculus. By Dr. H. G. Mudd, of St. Louis, Mo.

16. On the Radical Cure of Stricture by Dilating Urethrotomy, and

17. Demonstration of a Perfected Evacuator and an Improvement in the Method of Removal of Débris from the Bladder. By Dr. F. N. Otis, of New York City, N. Y.

18. Pyæmia as a Direct Sequel of Gonorrhœa. By Dr. R. Park, of Buffalo, N. Y.

19. Retrojections in Gonorrhœa. By Dr. E. R. Palmer, of Louisville, Ky.

20. Prostatotomy for Enlarged Prostate at the age of Forty-two. By Dr. Abner Post, of Boston, Mass.

21. A case of Removal of both Testicles for Recurrent Carcinoma, and

22. A Case of Nephrolithiasis Complicated with Hydronephrosis, in which Lumbar Nephrotomy was Performed. By Dr. F. W. Rockwell, of Brooklyn, N. Y.

23. Some Points on the Differential Diagnosis of Bladder and Kidney Affections, with Demonstrations of the Cystoscope and Other Instruments, and

24. On the Physiology of the Bladder. By Dr. Alexander W. Stein, of New York City, N. Y.

25. Local Treatment of Chronic Urethral Discharges. By Dr. F. R. Sturgis, of New York City, N. Y.

26. Some Points on the Etiology of Stricture of the Uretha. By Dr. R. W. Taylor, of New York City, N. Y.

27. Operative Treatment of Hypertrophy of the Prostate, and

28. Spontaneous Fracture of Stone in the Bladder. By Dr. F. S. Watson, of Boston, Mass.

29. The Relation of the Prostate to Chronic Urethal Discharges, and

The Value of the Tolerance of the Iodides as a Diagnostic of Syphilis, and

30. Urethral Stricture and Enlarged Prostate in their Relation to Vesical Calculus and Calculus Pyelitis, with cases. By Dr. J. William White, of Philadelphia, Pa.

BY INVITED GUESTS.

31. The Prognosis of Stricture, based on thirty years' death record of Stricture at the London Hospital and the practice at St. Peter's Hospital. By Dr. E. Hurry Fenwick, of London, England.

32. The Congenital Anomalies of the External Urethral Orifice. By Dr. C. Kaufmann, Zurich, Switzerland.

R. W. TAYLOR, *Secretary.*

---

A STATE SANITARY CONVENTION, under the auspices of *The State Board of Health and Vital Statistics of the Commonwealth of Pennsylvania*, will be held at Lewisburg, Penn., on the 17th and 18th days of May.

The Thirty-ninth Annual Meeting of *The American Medical Association* will be held in Cincinnati on Tuesday, May 8, and close on Friday, May 11.

The indications are that the meeting will be a very interesting one.

It is officially announced that the railroads will sell to physicians, and members of their families attending the meeting with them, round-trip tickets for one fare and one-third, as has been customary heretofore.

---

SUBSCRIPTIONS FOR THE RUSH MONUMENT FUND.—Dr. S. J. JONES, Argyle Building, Chicago, is the representative, for the State of Illinois, on the Committee of the American Medical Association, to solicit contributions towards the erection of a monument in honor of Dr. Rush. Donations of any amount may be sent to Dr. Jones, at the above address, or to Dr. G. H. Rohe, secretary, Baltimore, Md., or to J. M. Toner, Treasurer, Washington, D. C.

Volume LVI.] MAY, 1888. [Number 5.

### The CHICAGO MEDICAL JOURNAL AND EXAMINER

EDITOR:
S. J. JONES, M.D., LL.D

COLLABORATORS:

CHICAGO:

PROFESSOR E. ANDREWS, M.D., LL.D.
PROFESSOR J. BARTLETT, M.D.
PROFESSOR W. T. BELFIELD, M.D.
PROFESSOR F. BILLINGS, M.D.
PROFESSOR W. H. BYFORD, A.M., M.D.
PROFESSOR L. CURTIS, A.M., M.D.
PROFESSOR N. S. DAVIS, JR., A.M., M.D.
PROFESSOR O. C. DEWOLF, A.M., M.D.
PROFESSOR E. C. DUDLEY, A.B., M.D.
PROFESSOR C. FENGER, M.D.
PROFESSOR J. N. HYDE, A.M., M.D.
PROFESSOR E. F. INGALS, A.M., M.D.
PROFESSOR F. S. JOHNSON, A.M., M.D.
PROFESSOR H. A. JOHNSON, M.D., LL D.
PROFESSOR C. W. PURDY, M.D.
PROFESSOR C. G. SMITH, A.M., M.D.
PROFESSOR E. WING, A.M., M.D.

MILWAUKEE, WIS.
PROFESSOR N. SENN, M.D., Ph. D.

WASHINGTON, D. C.:
S. O. RICHEY, M.D.

UNITED STATES ARMY:
SURGEON D. L. HUNTINGTON, M.D.

UNITED STATES NAVY
SURGEON GENERAL J. M. BROWNE, M.D.
MEDICAL DIRECTOR A. L. GIHON, A.M., M.D

MARINE HOSPITAL SERVICE:
SURGEON S. T. ARMSTRONG, M.D., Ph.D.]

RAND, MCNALLY & COMPANY, PRINTERS.

1888.

